# Early Prediction of Postoperative Peritoneal Dialysis Using Lung Ultrasound Scoring in Neonates After Cardiopulmonary Bypass

**DOI:** 10.3390/jcdd13030121

**Published:** 2026-03-06

**Authors:** Duygu Tunçel, Süleyman Geter, Leyla Şero, Yiğit Kılıç, Nilüfer Okur, Bedri Aldudak

**Affiliations:** 1 Division of Neonatology, Department of Pediatrics, SBU Gazi Yaşargil Training and Research Hospital, 21010 Diyarbakır, Turkey; nilufer.maturokur@sbu.edu.tr; 2 Department of Pediatrics, SBU Gazi Yaşargil Training and Research Hospital, 21010 Diyarbakır, Turkey; sgeter21@gmail.com (S.G.); leyla.sero@saglik.com (L.Ş.); 3 Department of Pediatric Cardiovascular Surgery, SBU Gazi Yaşargil Training and Research Hospital, 21010 Diyarbakır, Turkey; dr.yigitkilic@gmail.com; 4 Department of Pediatric Cardiology, Diyarbakır Gazi Yaşargil Training and Research Hospital, 21010 Diyarbakir, Turkey; bedri.aldudak@saglik.gov.tr

**Keywords:** lung ultrasound, cardiopulmonary bypass, neonatal cardiac surgery, peritoneal dialysis, acute kidney injury, fluid overload, pulmonary edema, neonatal intensive care

## Abstract

Background: Neonates and young infants undergoing cardiac surgery with cardiopulmonary bypass (CPB) are highly vulnerable to pulmonary dysfunction, systemic inflammation, capillary leak, and fluid overload, which may lead to acute kidney injury (AKI) and the need for peritoneal dialysis (PD). Lung ultrasound (LUS) is a bedside, radiation-free tool that allows real-time assessment of lung aeration and pulmonary congestion. However, its role in predicting postoperative renal support remains limited. This study aimed to evaluate whether early postoperative LUS scores could predict the need for PD in neonates after congenital heart surgery with CPB. Methods: In this prospective single-center study, 53 neonates undergoing cardiac surgery with CPB between June 2025 and January 2026 were included. LUS was performed preoperatively and at 0–2 h, 24–48 h, 72 h, 120 h, and 168 h postoperatively using a standardized six-zone scoring system (0–18). The primary outcome was postoperative PD requirement. ROC analysis assessed predictive performance, and multivariable logistic regression identified independent predictors. Results: Total LUS scores significantly increased in the early postoperative period, remained elevated for 24–72 h, and gradually declined by days 5–7. Infants requiring PD (*n* = 16) had significantly higher LUS scores at 0–2 h, 24–48 h, and 72 h (*p* < 0.05). The 24–48 h (AUC = 0.784; sensitivity 87%, specificity 62% at cut-off ≥ 11.5) LUS score showed the best predictive value for PD (AUC = 0.831; sensitivity 86%, specificity 74% at cut-off ≥ 13). In multivariable analysis, higher LUS scores at 0–2 h (OR 1.625, *p* = 0.048) and 24–48 h (OR 1.621, *p* = 0.048) independently predicted PD. Conclusion: Postoperative LUS is a reliable, noninvasive tool that can aid in predicting the need for PD in neonates undergoing cardiac surgery with CPB, supporting timely fluid and renal management.

## 1. Introduction

After congenital heart surgery performed under cardiopulmonary bypass (CPB) in neonates and infants, the intense release of inflammatory mediators and ischemia-reperfusion injury that develops in the lungs can lead to structural and functional impairments of pulmonary tissue [1,2]. The systemic inflammatory response associated with CPB results in increased extravascular lung fluid due to increased capillary permeability, leakage of fluid into the third space, and the development of pulmonary edema. The inability to reliably quantify pulmonary edema by non-invasive methods forces clinicians to strike a difficult balance in postoperative fluid management between overloading the patient with fluids and restricting fluids, which can further worsen low cardiac output [3,4,5,6,7].

In recent years, observational studies have suggested that fluid overload in patients undergoing congenital heart surgery is not only a hemodynamic issue but can also serve as an early indicator of poor clinical outcomes [8,9]. A multicenter neonatal cohort has demonstrated the relationship between postoperative fluid accumulation and ventilation duration, intensive care unit length of stay, and other clinical outcomes [8]. Another study reported that fluid overload, assessed by weight measurement after congenital heart surgery, may be a prognostic factor for mortality and morbidity. These data suggest that early recognition of fluid overload may be clinically relevant in identifying patients at risk of prolonged respiratory support and Neonatal Intensive Care Unit (NICU) length of stay.

To prevent postoperative fluid overload in this high-risk patient group, some centers place a peritoneal catheter during cardiac surgery to enable prophylactic peritoneal dialysis (PD) or passive peritoneal drainage. However, in the literature, there is no consensus regarding the impact of this approach on clinical outcomes. To date, studies evaluating prophylactic PD or peritoneal drainage after heart surgery in neonates are limited in number, with the vast majority being single-center and observational in nature; only one single-center randomized controlled trial exists in the literature. Reported outcomes of prophylactic PD have been heterogeneous, with some studies suggesting improved fluid balance and clinical parameters, whereas others have not demonstrated consistent benefit [10,11,12,13,14,15,16,17].

Lung ultrasonography (LUS) has increasingly been used in recent years as a reliable imaging modality that allows bedside, real-time assessment of lung aeration in neonates without ionizing radiation [18]. LUS is effective in the early diagnosis of postoperative pulmonary complications such as pulmonary edema, atelectasis, and pneumothorax, and has assumed an important place in neonatal and pediatric intensive care practice [19]. Pulmonary dysfunction developing after neonatal cardiac surgery emerges as a result of the interaction between multiple factors, such as CPB-associated lung injury, fluid overload, systemic inflammatory response, and prolonged mechanical ventilation [20]. Therefore, early identification of pulmonary dysfunction is critically important for understanding disease severity and guiding clinical management.

While conventional chest X-rays have notable limitations such as radiation exposure and inadequate reflection of dynamic pulmonary changes, the ability of LUS to enable serial and repeatable evaluations provides a distinct advantage in clinical practice [21]. It should be noted that LUS does not distinguish between cardiogenic pulmonary congestion, capillary leak-related edema, or atelectasis; rather, it provides a semiquantitative assessment of global loss of lung aeration [22]. However, data regarding the prognostic value of LUS scores in the postoperative period following congenital heart surgery in neonates remains limited. Clarifying the relationship between postoperative LUS findings and mortality, length of hospital stay, and need for respiratory support, in particular, could significantly contribute to the management of this patient group.

It is known that the systemic inflammatory response developing after cardiopulmonary bypass, and pulmonary congestion due to capillary leakage and fluid overload, may coexist with renal perfusion impairment and acute kidney injury. In this context, the potential role of quantitatively assessed loss of lung aeration by lung ultrasonography as an early indicator of postoperative fluid imbalance and organ dysfunction, and as a predictor of the need for peritoneal dialysis, is attracting increasing attention [23].

The aim of this study is to assess whether systemic capillary leakage and fluid overload developing in the early post-CPB period can be objectively detected through lung aeration loss measured by LUS, and to evaluate its association with subsequent need for PD.

## 2. Materials and Methods

### 2.1. Study Design and Setting

This prospective, single-center study was conducted in the level 4 NICU of the Pediatric Cardiovascular Surgery Center between June 2025–January 2026. Approval was obtained from the local clinical research and ethics committee (date: 9 May 2025; number: 455). Verbal and written consent were obtained from the legal guardians of the patients included in the study.

### 2.2. Inclusion and Exclusion Criteria

Inclusion criteria: Term and preterm infants up to 44 weeks postconceptional age who underwent surgery for congenital heart disease and received CPB were included in the study (aged 0–28 days).

Exclusion criteria: Patients with major lung anomalies (pulmonary hypoplasia, congenital diaphragmatic hernia), those who were transferred to the NICU with extracorporeal membrane oxygenation (ECMO) after surgery, those who died within the first 24 h postoperatively, and those with incomplete data were excluded from the analysis.

All consecutive neonates meeting inclusion criteria were enrolled.

### 2.3. Perioperative Clinical Data Collection

Demographic and natal data, including birth weight (grams), gestational age (weeks), mode of delivery (cesarean section (C/S) or normal vaginal delivery (NVD)), and 5th minute Apgar score, were recorded. Cardiac diagnosis and surgical characteristics included postnatal surgery timing (days), RACHS-1 (risk adjustment for congenital heart surgery), CPB duration (min), and aortic cross-clamp duration (min).

Postoperative clinical parameters included: the highest vasoactive-inotropic score (VIS) in the first 24 h postoperatively (calculated as dopamine (µg/kg/min) + dobutamine (µg/kg/min) + 100 × epinephrine (µg/kg/min) + 10 × milrinone (µg/kg/min) + 10,000 × vasopressin (U/kg/min) + 100 × norepinephrine (µg/kg/min)); duration of invasive mechanical ventilation (days); duration of noninvasive mechanical ventilation (days); length of hospital stay (days); mortality; and requirement for peritoneal dialysis.

### 2.4. Definition of Clinical Outcomes

In neonates undergoing cardiovascular surgery, PD was initiated in the postoperative period. The PD was used at the discretion of the clinical teams using the following general criteria: if there was fluid overload ≥10% calculated using cumulative fluid balance (FO (%) = (total fluid in − total fluid out)/admission weight × 100); oliguria (<1 mL/kg/h for 6 h) despite diuretics; rising serum creatinine with metabolic acidosis; or hemodynamic instability with inadequate response to medical therapy. In addition, the inability to achieve volume control in the presence of refractory metabolic acidosis, hyperkalemia, and low cardiac output syndrome were accepted as additional indications for PD. The clinician performing LUS was not involved in decisions regarding peritoneal dialysis initiation. PD decisions were made independently by the attending intensivist based on clinical and laboratory parameters. LUS findings were not incorporated into the formal PD initiation protocol during the study period.

### 2.5. Lung Ultrasound Protocol

All patients underwent lung ultrasonography by a single neonatal intensive care specialist with formal training in lung ultrasonography and extensive experience. The time points at which lung ultrasound was performed were one day before the operation, at 0–2, 24–48, and 72 ± 6 h, and on days 5 and 7 postoperatively, until the patient was extubated. Inter-observer variability was not formally assessed, which represents a limitation of the study.

**Figure 1:**
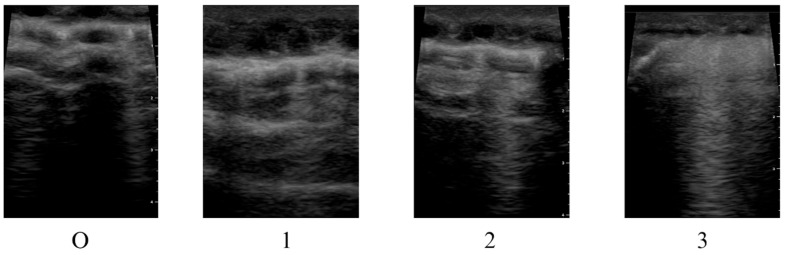
Lung ultrasound score used during study protocol. Representative ultrasound images corresponding to lung ultrasound scores: 0 = normal aeration (A-line pattern), 1 = presence of discrete B-lines, 2 = confluent B-lines or “white lung” appearance, and 3 = lung consolidation.

Lung ultrasonography was performed using an ultrasound system (GE Healthcare, Chicago, IL, USA) with a high-frequency linear transducer (8 MHz) evaluating a total of six regions, three on each lung. Each region was scored from 0 to 3 points according to the degree of lung aeration loss: 0 points for normal aeration (A-line pattern), 1 point for the presence of discrete B-lines, 2 points for confluent B-lines or a “white lung” appearance, and 3 points for the presence of consolidation (Figure 1). The total lung ultrasound score ranges from 0 to 18. For clinical evaluation, the total LUS was also classified as mild (0–6), moderate (7–12), and severe (13–18) aeration loss [24].

### 2.6. Postoperative Management Protocol

In postoperative fluid management, we aimed to achieve an early negative fluid balance by closely monitoring patients (daily fluid balance, weight change, urine output) to avoid a positive fluid balance due to capillary leakage, inflammation, and impaired kidney function. In general, we started with 75–80% of the fluid required in the postoperative period and continued with early enteral feeding according to the patient’s clinical condition. Furosemide (0.1–0.4 mg/kg/hour continuous infusion) was routinely initiated in all patients during the postoperative period, with the aim of achieving the targeted negative fluid balance by supporting early diuresis, especially in the first 24–48 h postoperatively. Pressure-controlled modes were preferred for invasive mechanical ventilation (assist-control or synchronized intermittent positive pressure ventilation). Early extubation was targeted as long as hemodynamics permitted, and lung-protective settings (low tidal volume, appropriate PEEP, avoiding unnecessarily high FiO_2_, recruitment when needed) were used to reduce the risk of atelectasis and potential progressive lung injury.

### 2.7. Outcomes

The main study outcome was the need for peritoneal dialysis; additional clinical endpoints were mechanical ventilator duration, VIS, mortality, and length of stay.

### 2.8. Statistical Analysis

All statistical analyses were performed using IBM SPSS Statistics for Windows, Version 27.0 (IBM Corp., Armonk, NY, USA).

The distribution of continuous variables was assessed using visual methods (histograms and Q–Q plots) and analytical tests (Kolmogorov–Smirnov and Shapiro–Wilk tests). Normally distributed variables were presented as mean ± standard deviation, whereas non-normally distributed variables were expressed as median (minimum–maximum or interquartile range, as appropriate). Categorical variables were summarized as numbers and percentages.

Comparisons between two independent groups (PD vs. non-PD) were performed using Student’s *t*-test for normally distributed variables and the Mann–Whitney U test for non-normally distributed variables. Categorical variables were compared using the chi-square test or Fisher’s exact test, as appropriate.

Serial postoperative lung ultrasound (LUS) measurements were analyzed at predefined time points. Group comparisons at each time point were conducted using appropriate parametric or non-parametric methods according to distributional assumptions.

To evaluate the diagnostic performance of postoperative LUS scores for predicting PD requirement, receiver operating characteristic (ROC) curve analysis was performed. The area under the curve (AUC) with 95% confidence intervals was calculated. Sensitivity, specificity, and optimal cut-off values were determined using the Youden index. Decision curve analysis (DCA) was performed to evaluate the clinical utility of the multivariable logistic regression models (including postoperative LUS score and CPB duration) for predicting peritoneal dialysis requirement by calculating net benefit across a range of threshold probabilities and comparing the model with treat-all and treat-none strategies. Given the limited number of PD events, multivariable logistic regression models were constructed using a parsimonious approach to reduce the risk of overfitting. Separate models were developed for each postoperative LUS time point. Only clinically relevant variables and those considered potential confounders were included in the models. Results were reported as odds ratios (OR) with 95% confidence intervals. Model fit was evaluated using the omnibus test, Hosmer–Lemeshow goodness-of-fit test, and Nagelkerke R^2^.

A two-sided *p* value < 0.05 was considered statistically significant for all analyses.

## 3. Results

This prospective study included a total of 53 newborn infants between 1 June 2025 and 25 January 2026. In the study cohort, the three most common primary diagnoses were transposition of the great arteries (24 patients, 45.2%), coarctation of the aorta (nine patients, 16.9%), and aortic arch hypoplasia (eight patients, 15.0%), which together accounted for the majority of cases. The distribution of the surgical procedures performed is presented in Table 1.

As shown in Table 2, the mean birth weight was 2861 ± 529 g, and the mean gestational age was 37.7 ± 1.5 weeks. Most infants were delivered by cesarean section (79.2%), and 52.8% were male. The median 5 min Apgar score was six (range, 1–9). The median postnatal age at surgery was 14 days (range, 4–78), with a mean body weight of 3570 ± 950 g and a mean postconceptional age of 40.6 ± 2.1 weeks at the time of surgery. The median RACHS-1 score was three (range, 2–6), indicating a moderate overall surgical risk profile in this cohort.

Peritoneal dialysis was performed in 16 patients (30%) during the postoperative period. In the postoperative period, the median time to initiation of peritoneal dialysis was 20 h (minimum 3–maximum 72). Among the 16 patients who required PD, none received PD within the first 0–2 h postoperatively. The total of eight patients (50%) initiated PD between 2 and 24 h, followed by four patients (25%) between 24 and 48 h, three patients (18.8%) between 48 and 72 h, and one patient (6.25%) at 72 h or later. These findings provide a detailed overview of the temporal pattern of PD initiation in this cohort and were incorporated into Section 3.

As presented in Table 3, LUS scores were compared between patients who required peritoneal dialysis (PD) and those who did not. Preoperative LUS scores were similar between the groups and did not reach statistical significance (*p* = 0.160).

However, in the early postoperative period, LUS scores were significantly higher in the PD (+) group. At 0–2 h postoperatively, the median LUS score was 16 (6–18) in the PD (+) group compared with 12 (6–18) in the PD (–) group (*p* = 0.016). Similarly, at 24–48 h and 72 h, LUS scores remained significantly higher in patients requiring PD (*p* = 0.032 for both time points).

Among the evaluated parameters, the postoperative vasoactive-inotropic score was significantly higher in the PD (+) group compared with the PD (−) group (median 17 (14.75–23) vs. 14 (12–17), *p* = 0.008). In addition, the duration of invasive mechanical ventilation was significantly longer in patients requiring PD (median 15 (8–21.75) days vs. 5 (3–8) days, *p* = 0.001).

Other variables, including birth weight, gestational age, RACHS score, cardiopulmonary bypass duration, aortic cross-clamp time, operation day, non-invasive ventilation duration, and length of hospital stay, did not differ significantly between the groups. These findings are presented in detail in Table 4.

In the multivariable logistic regression analysis predicting the requirement for peritoneal dialysis, only the LUS score at the relevant time point and CPB duration were included together in each model. In all three models, the LUS score was independently associated with the need for peritoneal dialysis (Model 1: OR = 1.308, 95% CI 1.030–1.662, *p* = 0.028; Model 2: OR = 1.262, 95% CI 1.045–1.525, *p* = 0.016; Model 3: OR = 1.200, 95% CI 1.031–1.396, *p* = 0.019).

In contrast, CPB duration was not statistically significant in any of the models (all *p* > 0.65). The overall fit of the models was statistically significant (omnibus test *p* < 0.05), and the Hosmer–Lemeshow test indicated good calibration (*p* > 0.75 for all models). Nagelkerke R^2^ values ranged between 20% and 24%, suggesting a moderate explanatory capacity of the models (Table 5).

**Figure 2:**
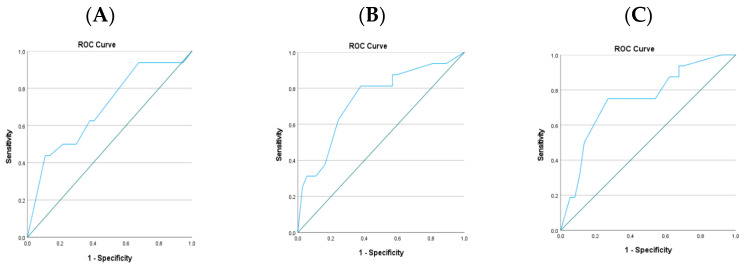
ROC curves of postoperative lung ultrasound scores at different time intervals. (**A**) postoperative 0–2 h LUS, (**B**) postoperative 24–48 h LUS, and (**C**) postoperative 72 h LUS.

Postoperative lung ultrasound scores demonstrated a significant ability to discriminate patients who required PD from those who did not. As shown in Figure 2, the area under the ROC curve (AUC) values indicated moderate diagnostic performance at all evaluated time points. The highest discriminative accuracy was observed at 24–48 h postoperatively (AUC: 0.784; 95% CI: 0.653–0.915; *p* = 0.001), followed closely by the 72 h measurement (AUC: 0.781; 95% CI: 0.646–0.916; *p* = 0.002). The early postoperative 0–2 h LUS score also showed significant predictive ability (AUC: 0.738; 95% CI: 0.595–0.881; *p* = 0.008), although its performance was comparatively lower.

As detailed in Table 6, the optimal cut-off values for predicting PD requirement were 13.5 at 0–2 h and 11.5 at both 24–48 h and 72 h. The 24–48 h LUS score yielded the highest sensitivity (87%) but moderate specificity (62%), suggesting strong screening capability during this interval. In contrast, the 72 h measurement demonstrated a more balanced profile with 80% sensitivity and 73% specificity. The 0–2 h LUS score showed modest sensitivity (67%) and specificity (63%), indicating that early LUS assessment has some predictive value.

**Figure 3:**
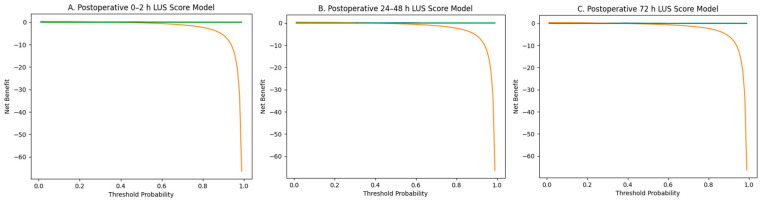
Decision curve analysis for prediction of peritoneal dialysis requirement. (**A**) Postoperative 0–2 h LUS score model. (**B**) Postoperative 24–48 h LUS score model. (**C**) Postoperative 72 h LUS score model.

Decision curve analysis was performed to evaluate the clinical utility of LUS scores at different postoperative time points for predicting PD requirement (Figure 3A–C).

At all time points, the multivariable model including LUS score and CPB duration demonstrated a higher net benefit than both the treat-all and treat-none strategies across clinically relevant threshold probability ranges.

The net benefit was more pronounced at low and intermediate threshold probabilities. The decrease in net benefit at higher threshold probabilities is consistent with the limited number of PD events in the cohort.

A total of eight patients (5%) died during the postoperative period: four patients (25%) in the peritoneal dialysis group and four patients (10.8%) in the non-dialysis group (*p* = 0.180).

Three deaths were attributed to sepsis-related multiple organ failure, four to capillary leak syndrome and low cardiac output syndrome, and one to hepatic failure and pulmonary hemorrhage.

## 4. Discussion

In this prospective study, the serial changes in LUS before and after surgery in neonates undergoing cardiopulmonary bypass, as well as their relationship with clinical outcomes, were evaluated in detail. Importantly, higher early postoperative LUS was consistently associated with subsequent PD requirements. ROC analyses demonstrated moderate discriminatory ability, particularly at 24–48 and 72 h postoperatively. In parsimonious multivariable models adjusted for cardiopulmonary bypass duration, LUS scores remained independently associated with PD requirement.

Previous studies have demonstrated that prolonged cardiopulmonary bypass and aortic cross-clamp durations are associated with enhanced systemic inflammatory response and pulmonary capillary leak, particularly in neonates. In one study, high scores on lung ultrasonography were found to be associated with long CPB [7]. CPB duration did not remain independently associated with PD requirement in multivariate models, suggesting that LUS may more closely reflect physiological effects related to surgical stress rather than bypass duration.

Studies on lung ultrasound following cardiac surgery have been designed to focus on comparing it with chest X-ray and predicting pulmonary complications [25,26]. There are also a small number of studies focusing on the inflammatory response and fluid overload occurring in newborns following CPB [27]. Diwakar et al. demonstrated that utilizing LUS to assess extravascular lung water content can reliably predict ventilatory outcomes in pediatric patients, reinforcing the need for LUS as an integral part of postoperative management [28]. Additionally, Song et al. [29] reported that patients who underwent LUS-guided recruitment maneuvers experienced shorter durations of mechanical ventilation compared to controls, indicating a direct impact on respiratory outcomes. Although not statistically significant, the findings of the same study indicated that the length of hospital stay was indirectly reduced in patients who underwent the opening maneuver using LUS. Additionally, patients requiring PD had significantly higher vasoactive-inotropic scores postoperatively and markedly longer invasive mechanical ventilation durations. These findings indicate that the need for PD is associated with greater hemodynamic instability and respiratory support requirements. The consistent relationship between higher LUS scores and prolonged ventilation further supports the ability of LUS to reflect clinically significant pulmonary dysfunction in this population. In contrast, LUS scores were not significantly associated with mortality in this cohort. Mortality rates were comparable between PD and non-PD groups. This finding suggests that while LUS may reflect pulmonary congestion and early fluid imbalance, its prognostic utility for survival outcomes in this relatively small cohort remains uncertain.

One of the noteworthy findings of the present study was that higher LUS scores in the early postoperative period were associated with the requirement for subsequent peritoneal dialysis (PD). PD practices vary among centers performing neonatal cardiac surgery; while some adopt prophylactic intraoperative catheter placement, others initiate PD postoperatively based on persistent oliguria, fluid overload, or biochemical evidence of acute kidney injury [30]. Neonates are particularly vulnerable to capillary leak syndrome and fluid overload following cardiopulmonary bypass, which may contribute to both pulmonary congestion and renal dysfunction. In our cohort, postoperative LUS scores at 0–2, 24–48, and 72 h were significantly higher in patients who subsequently required PD. The 24–48 h measurement demonstrated the highest discriminatory performance (AUC 0.784), with the 72 h score showing comparable accuracy. Although the 0–2 h assessment also showed significant predictive ability, its performance was relatively lower. These findings suggest that LUS may provide clinically relevant information during the early postoperative window, when fluid redistribution and systemic capillary leak are most pronounced. In multivariable models restricted to LUS and cardiopulmonary bypass duration to reduce the risk of overfitting, LUS remained independently associated with PD requirement. However, given the limited number of events and the absence of external validation, these findings should be interpreted as hypothesis-generating rather than definitive evidence of predictive superiority.

This study has significant limitations. Firstly, due to the limited sample size and number of PD events, statistical power and the ability to fully adjust for potential confounding factors were constrained. Given the limited number of PD events, multivariable models were restricted to LUS and CPB duration to reduce the risk of overfitting. Secondly, this study was conducted at a single center, using LUS by an experienced operator; therefore, inter-observer variability and generalizability require further evaluation. The predictive performance of LUS was evaluated within the study cohort, and external validation in independent populations is warranted. Finally, the severity of acute kidney injury according to KDIGO criteria was not systematically included in the analysis, particularly among patients who did not receive PD.

## 5. Conclusions

In conclusion, this study suggests a possible association between early postoperative LUS scores and PD requirement in neonates following CPB. However, given the single-center design, limited sample size, and low number of events, the findings should be considered hypothesis-generating rather than definitive evidence. The integration of LUS into clinical decision-making requires larger, multicenter studies with external validation. The present data may serve as a promising basis for more comprehensive future research in this field.

## Figures and Tables

**Table 1:** Primary procedures performed on patients.

	Number of Patients (*n* = 53)
Arterial switch operation, *n* (%)	24 (45.2)
Coarctation repair, *n* (%)	9 (16.9)
Aortic arch repair, *n* (%)	8 (15)
Norwood procedure, *n* (%)	3 (5.6)
Truncus repair, *n* (%)	3 (5.6)
Pulmonary banding/mBTT shunt, *n* (%)	2 (3.76)
Total anomalous pulmonary venous return repair, *n* (%)	1 (1.88)
Tetralogy of Fallot repair, *n* (%)	1 (1.88)
Ventricular septal defect closure, *n* (%)	1 (1.88)
Arterial switch + VSD closure, *n* (%)	1 (1.88)

mBTT: modified Blalock-Taussig-Thomas, VSD: ventricular septal defect.

**Table 2:** Selected demographic and clinical characteristics of the patients.

Variable	Number of Patients (*n* = 53)
Birth weight, g *	2861 ± 529
Gestational age, weeks *	37.7 ± 1.5
Mode of delivery, C-section, *n* (%)	42 (79.2)
Male sex, *n* (%)	28 (52.8)
5 min Apgar score **	6 (1–9)
Postnatal age at surgery, days **	14 (4–78)
Weight at the time of surgery, g *	3570 ± 950
Postconceptional age at the time of surgery, weeks *	40.6 ± 2.1
RACHS-1 score **	3 (2–6)

* Mean ± standard deviation, ** Median (minimum–maximum), RACHS-1: Risk Adjustment for Congenital Heart Surgery.

**Table 3:** Comparison of LUS scores according to need for peritoneal dialysis and prolonged mechanical ventilation.

Time Point *	PD (+) (*n* = 16)	PD (−) (*n* = 37)	*p* Value
Preoperative LUS score	8 (0–15)	6 (0–14)	0.160
Postoperative 0–2 h LUS score	16 (6–18)	12 (6–18)	0.016
Postoperative 24–48 h LUS score	14 (2–18)	10 (2–18)	0.032
Postoperative 72 h LUS score	13 (2–18)	9.5 (0–18)	0.032
Postoperative 120 h LUS score	12 (7–18)	7 (0–12)	0.068
Postoperative 168 h LUS score	11 (8–12)	7 (0–18)	0.159

* Median (minimum–maximum).

**Table 4:** Comparison of clinical variables according to peritoneal dialysis requirement.

Variable	PD (+) (n = 16)	PD (−) (*n* = 37)	*p*
Birth weight (g) *	3000 (2625–3297.5)	2840 (2600–3052.5)	0.393
Gestational age (weeks) **	38 (37–38)	38 (36.75–39)	0.833
RACHS score **	3.00 (3.00–3.00)	3.00 (3.00–3.00)	0.165
CPB duration (min) *	169.36 ± 59.20	150.16 ± 57.18	0.319
Aortic cross-clamp time (min) *	110.36 ± 41.00	92.88 ± 46.60	0.210
Operation day **	10 (8–15.5)	18 (10–30)	0.124
Postoperative VIS score **	17 (14.75–23)	14 (12–17)	**0.008**
Invasive ventilation (days) **	15 (8–21.75)	5 (3–8)	**0.001**
Non-invasive ventilation (days) **	3 (0.75–8)	1 (1–4)	0.527
Length of hospital stay (days) **	30 (17.5–42)	28 (15–34)	0.620

* Mean ± standard deviation, ** Median (IQR 25–75). CPB, cardiopulmonary bypass; RACHS, Risk Adjustment for Congenital Heart Surgery; VIS, Vasoactive-Inotropic Score; IQR, interquartile range. Bold *p*-values indicate statistical significance (*p* < 0.05).

**Table 5:** Multivariable logistic regression models for prediction of peritoneal dialysis requirement.

Model	Variable	β	OR (95% CI)	*p*
Model 1 (Postoperative 0–2 h)	LUS score	0.269	1.308 (1.030–1.662)	0.028
	CPB duration (min)	−0.001	0.999 (0.986–1.012)	0.867
Model 2 (Postoperative 24–48 h)	LUS score	0.233	1.262 (1.045–1.525)	0.016
	CPB duration (min)	0.001	1.001 (0.988–1.014)	0.914
Model 3 (Postoperative 72 h)	LUS score	0.182	1.200 (1.031–1.396)	0.019
	CPB duration (min)	0.003	1.003 (0.990–1.016)	0.650

Model fit statistics: Model 1: Omnibus χ^2^(2) = 7.248, *p* = 0.027; Nagelkerke R^2^ = 0.209; Hosmer–Lemeshow *p* = 0.755; Model 2: Omnibus χ^2^(2) = 8.436, *p* = 0.015; Nagelkerke R^2^ = 0.241; Hosmer–Lemeshow *p* = 0.773; Model 3: Omnibus χ^2^(2) = 7.800, *p* = 0.020; Nagelkerke R^2^ = 0.224; Hosmer–Lemeshow *p* = 0.798; LUS, lung ultrasound score; CPB, cardiopulmonary bypass.

**Table 6:** Diagnostic performance of postoperative LUS scores for peritoneal dialysis requirement.

	Cut-Off Level	AUC	Lower Bound	Upper Bound	Sensitivity (%)	Specificity (%)	*p*
Postoperative 0–2 h LUS score	13.5	0.738	0.595	0.881	67	63	0.008
Postoperative 24–48 h LUS score	11.5	0.784	0.653	0.915	87	62	0.001
Postoperative 72 h LUS score	11.5	0.781	0.646	0.916	80	73	0.002

## Data Availability

The datasets generated and analyzed during the current study are not publicly available due to the inclusion of sensitive personal health information and restrictions arising from the national Personal Data Protection Law. The data contain identifiable and potentially re-identifiable patient information from neonates, and therefore cannot be shared in open-access repositories. However, the data may be made available from the corresponding author upon reasonable request and in accordance with ethical approval, institutional policies, and legal requirements, including review by the journal editors when deemed necessary.

## Abbreviations

The following abbreviations are used in this manuscript:

CPB	Cardiopulmonary Bypass
AKI	Acute Kidney Injury
PD	Peritoneal Dialysis
LUS	Lung Ultrasonography
NICU	Neonatal Intensive Care Unit
ECMO	Extracorporeal Membrane Oxygenation
C/S	Cesarean Section
NVD	Normal Vaginal Delivery
RACHS	Risk Adjustment for Congenital Heart Surgery
VIS	Vasoactive-Inotropic Score
PEEP	Positive End-Expiratory Pressure
FiO_2_	Fraction of Inspired Oxygen
ROC	Receiver Operating Characteristic
AUC	Area Under the Curve
OR	Odds Ratio
SPSS	Statistical Package for the Social Sciences
